# Preregistration of high‐quality protocols in pharmacoepidemiology research

**DOI:** 10.1002/jcv2.70020

**Published:** 2025-06-07

**Authors:** Henrik Larsson, Zhang Chang, Kenneth K. C. Man

**Affiliations:** ^1^ School of Medical Sciences Örebro University Örebro Sweden; ^2^ Department of Medical Epidemiology and Biostatistics Karolinska Institutet Stockholm Sweden; ^3^ Research Department of Practice and Policy UCL School of Pharmacy London UK

**Keywords:** child and adolescent mental health, open science, pharmacoepidemiology, preregistration, Registered Reports, reproducible research

## Abstract

Pharmacoepidemiology studies are an important complement to Randomized Clinical trials, but such studies face several challenges, such as confounding and selective reporting. How to best address confounding has been discussed in detail for many years. More recent discussions have highlighted the value of pharmacoepidemiology studies based on pre‐registered protocols. This is an important step to address problems related to selective reporting and to enhance transparency and reproducibility. In this editorial perspective, we discuss the value of pre‐registered protocols in pharmacoepidemiology.

## EDITORIAL PERSPECTIVE

One critical goal in child and adolescent mental health research is to build a robust evidence base around treatment effectiveness and safety. Addressing questions like ‘Which pharmacological treatments work?’ is vital, as effective interventions can make a significant impact on young people's lives, shaping their development and future well‐being. While Randomized Clinical trials (RCT) are the gold standard for establishing efficacy and safety, they are often conducted in controlled settings, which limits their generalizability to real‐world populations (Garcia‐Argibay et al., 2025; Wong et al., 2019). RCTs typically involve selective patient populations and focus on specific outcomes within a short follow‐up period, potentially overlooking the complexities in routine clinical care. These limitations may be particularly relevant in child and adolescent mental health settings, where individual characteristics vary widely, and multimorbidity is common.

The need for high‐quality pharmacoepidemiology studies, as a complement to RCTs, has been increasingly recognized in child and adolescent mental health research and beyond (Garcia‐Argibay et al., 2025). By leveraging Real‐World Daa (RWD) sources from large‐scale health registers, electronic health record databases, and other sources of observational data, pharmacoepidemiology can provide insights into real‐world effects of pharmacotherapy across a broader spectrum of patients, outcomes, settings and timeframes than those typically included in RCTs.

An important strength of recent child and adolescent pharmacoepidemiology research is the increasing availability of RWD sources. A recent study conducted a review of 55 globally available RWD sources that can be used for child and adolescent pharmacoepidemiologic research. The study reported that most RWD sources were available in Europe and the United States, but also in Asia and the Pacific area (Wharton et al., 2024). The increasing availability of RWD sources for pharmacoepidemiology research across the globe could build up for multi‐country studies. This is important given that most of the previous pharmacoepidemiology research in child and adolescent settings have used data from one country only (Wong et al., 2019). Combined use of RWD from several countries with different health care systems is critical to examine the external validity and generalisability of results of pharmaco‐epidemiological studies.

In contrast to RCTs, that use treatment assignment to help balance potential confounding factors (i.e., measured or unmeasured), pharmacoepidemiology studies are prone to bias, if confounding is not properly addressed. Pharmacoepidemiology studies, therefore, need to address confounding carefully to avoid biased findings that could have serious negative implications for research, clinics, patients and society (e.g., miss‐directed treatment recommendations). A particularly important type of confounding in pharmacoepidemiology studies is confounding by indication, which occurs when factors involved in selecting patients for a particular treatment also affect the outcome (Kyriacou & Lewis, 2016). Several studies have recently described how confounding can be addressed using different study designs and statistical approaches (Chang et al., 2019; Sendor & Stürmer, 2022; Wong et al., 2019). Within‐individual designs, or self‐controlled designs, present one approach to account for confounding by indication (Chang et al., 2019). Instead of comparing medication users and nonusers, within‐individual designs compare the risk of outcomes during medicated time periods with nonmedicated time periods within the same individual. Because the comparison is within the same individual, all confounding from factors that are stable throughout the observed time at risk is eliminated, even if the factors are unmeasured or unknown (Chang et al., 2019).

Another concern in pharmacoepidemiology research, which has been articulated in recent reviews and editorials, concerns methodological transparency and selective reporting (The European Network of Centres for Pharmacoepidemiology and Pharmacovigilance, n.d.; Wang & Pottegård, 2024). First, non‐significant studies may not be published at all, which may lead to publication bias. Second, even when studies are published, findings may be selectively reported to prioritize statistically significant or favoured results over nonsignificant results. Selective reporting is a general problem in the research process. To counter it, researchers undertaking RCTs are now obliged to publicly preregister study protocols, setting out the rationale for the study, the key research questions, the inclusion and exclusion criteria, the primary outcome variables, and the statistical analysis plan. Today, most publications that report findings from systematic reviews are also based on pre‐registration of study protocols. Authorities such as the European Medicines Agency (EMA), the International Society for Pharmacoepidemiology (Public Policy Committee, International Society of Pharmacoepidemiology, 2016), and the European Network of Centres for Pharmacoepidemiology and Pharmacovigilance (ENCePP) all actively recommend pre‐registration of observational studies, particularly in pharmacoepidemiology, to increase transparency, reduce bias, and strengthen real‐world evidence. As part of the EMA and Heads of Medicines Agencies (HMA) Big Data Initiative, the Catalogue of RWD Sources and the Catalogue of RWD Studies were introduced in 2024 (https://catalogues.ema.europa.eu/catalogue‐rwd‐sources). These new catalogues help regulators, researchers, and pharmaceutical companies to identify relevant real‐world data sources and studies, and to support research based on FAIR principles (Findable, Accessible, Interoperable, and Reusable). In addition to the EMA/HMA catalogues, pharmacoepidemiology study protocols and reports can also be registered on other platforms, such as the Open Science Forum (https://osf.io/).

## THE VALUE OF PRE‐REGISTERED PROTOCOLS IN PHARMACOEPIDEMIOLOGY

Pre‐registration offers significant value in pharmacoepidemiology research by providing a public record of a study's planned hypotheses, methods, and analysis strategy before the data are collected or analysed (Wang & Pottegård, 2024), which helps to mitigate selective reporting. In the context of pharmacoepidemiology, where large datasets offer many possibilities for subgroup analyses and exploratory findings, pre‐registration serves as a protection against these risks by committing researchers to a pre‐specified plan. This commitment improves the credibility of the research, as it demonstrates that findings were not driven by post hoc adjustments or data‐driven discovery alone. Pre‐registration also fosters greater transparency and accountability in the research process, allowing other researchers and the public to scrutinize any deviations from the original plan. By promoting these standards, pre‐registration ultimately strengthens the reliability and integrity of pharmacoepidemiology research, building trust in its findings as a source of real‐world evidence to inform clinical and policy decisions (Wang & Pottegård, 2024).

Pre‐registered protocols are also beneficial for multi‐site collaborations as it ensures that all collaborators adhere to the same study design—a crucial factor in complex pharmacoepidemiology studies where data harmonization is essential for pooling data from diverse sources. Beyond serving technical purposes, a well‐defined protocol fosters a mutual understanding of the study conduct, terminology, and regional variations among participating sites, thereby streamlining collaborative efforts.

While pre‐registration provides a robust framework for reproducibility, it does not preclude necessary modifications. Deviations from the original study protocol are possible and sometimes necessary. Changes may arise due to new data availability, methodological improvements, regulatory feedback, or unforeseen challenges in data collection or analysis. However, any deviation must be justified transparently to maintain the study's credibility. Any modifications must be clearly documented, scientifically justified, time‐stamped, and reported in the final publications. While pre‐registration ensures reproducibility and credibility, a well‐documented justification process allows flexibility without compromising research integrity.

## INCREASE PROTOCOL QUALITY

While some form of protocol may be used by many investigators, there is a great deal of variation in quality (Wang et al., 2023). Further, not all groups within pharmacoepidemiology research have established a practice of finalizing study protocols, such as documentation of subsequent amendments. Protocol quality can be raised by using protocol templates that incorporate pharmacoepidemiology good practice guidance. There are several existing protocol templates that could be used for this purpose. For example, the HARmonized Protocol Template to Enhance Reproducibility provides a set of core recommendations for clear and reproducible pharmacoepidemiology study protocols and is intended to be used as a backbone throughout the research process from developing a valid study protocol, to registration, through implementation and reporting on those implementation decisions (Wang et al., 2023). In addition to using template‐based approaches, engaging in internal and external peer reviews of the protocol could improve the quality of the protocol. Collaborative review sessions among investigators or with external experts can help identify ambiguities, omissions, or methodological issues early in the research process. In particular, involving relevant stakeholders—including clinicians, data scientists, and patient representatives—to ensure that the protocol addresses practical concerns and is aligned with real‐world needs.

## CONCLUSIONS AND FUTURE DIRECTIONS

Pharmacoepidemiology studies are an important complement to RCTs, but such studies face several challenges, such as confounding and selective reporting. How to best address confounding has been discussed in detail for many years. More recent discussions have highlighted the value of pharmacoepidemiology studies based on pre‐registered protocols. This is an important step to address problems related to selective reporting and to enhance transparency and reproducibility. Based on these recent discussions, we would like to suggest the following good practices that we encourage research teams to follow, when preparing pharmacoepidemiology studies to JCPP Advances or other journals.

- Pre‐register study protocols and/or conduct research as a Registered Report, to help protect against questionable research practices and selective reporting. Indeed, JCPP Advances are interested in research as Registered Reports (Baldwin and Larsson, 2024).
- When preparing the protocol, use protocol templates that incorporate pharmacoepidemiology good practice guidance.
- Consider using relevant reporting guidelines such as the “Reporting of studies conducted using observational routinely collected health data statement for pharmacoepidemiology” (RECORD‐PE).

## AUTHOR CONTRIBUTIONS

**Henrik Larsson**: Conceptualization; methodology; writing—original draft. **Zhang Chang**: Writing—review and editing. **Kenneth K. C. Man**: Writing—review and editing.

## CONFLICT OF INTEREST STATEMENT

Henrik Larsson reports receiving grants from Shire Pharmaceuticals; personal fees from and serving as a speaker for Shire Pharmaceuticals and Evolan Pharma AB outside the submitted work; and sponsorship for a conference on attention‐deficit/hyperactivity disorder from Shire Pharmaceuticals outside the submitted work. Henrik Larsson is Editor in Chief of JCPP Advances. Z.C. received speaker fees from the Takeda Pharmaceuticals, outside the submitted work. K.K.C.M. reports receiving grants from the CW Maplethorpe Fellowship, the European Union Horizon 2020, the UK National Institute of Health Research, the Hong Kong Research Grant Council, the Hong Kong Innovation and Technology Commission, and the Korea Ministry of Food and Drug Safety, outside the submitted work.

## ETHICAL CONSIDERATIONS

Not applicable.

## Data Availability

Data sharing not applicable to this article as no datasets were generated or analysed during the current study.
